# Monoacylglycerol lipase promotes progression of hepatocellular carcinoma via NF-κB-mediated epithelial-mesenchymal transition

**DOI:** 10.1186/s13045-016-0361-3

**Published:** 2016-11-25

**Authors:** Weiping Zhu, Yiming Zhao, Jiamin Zhou, Xin Wang, Qi Pan, Ning Zhang, Longrong Wang, Miao Wang, Dihua Zhan, Zeyang Liu, Xigan He, Dening Ma, Shuang Liu, Lu Wang

**Affiliations:** 1 Department of Hepatic Surgery, Fudan University Shanghai Cancer Center, Shanghai Medical College, Fudan University, Shanghai, 200032 People’s Republic of China; 2 Department of Rheumatology, Xiyuan Hospital, China Academy of Chinese Medical Sciences, 1 Xiyuan Caochang, Haidian District, Beijing, 100091 People’s Republic of China; 3 Liver Cancer Institute, Zhongshan Hospital, Fudan University, Shanghai, 200032 People’s Republic of China

**Keywords:** MAGL, Hepatocellular carcinoma, NF-κB, EMT

## Abstract

**Background:**

Monoacylglycerol lipase (MAGL), a critical lipolytic enzyme, has emerged as a key regulator of tumor progression, yet its biological function and clinical significance in hepatocellular carcinoma (HCC) is still unknown.

**Methods:**

In this study, we used a tissue microarray containing samples from 170 HCC patients to evaluate the expression of MAGL and its correlation with other clinicopathologic characteristics. In addition, we investigated the regulating effects of MAGL on various HCC lines. Finally, we identified the NF-κB signaling pathway participated in MAGL-mediated epithelial-mesenchymal transition (EMT) using HCC cell lines with different metastatic potentials.

**Results:**

The expression of MAGL was significantly higher in HCC tumors than in matched peritumor tissues. Specifically, high MAGL expression was found in tumors with larger tumor size, microvascular invasion, poor differentiation, or advanced TNM stage. In addition, the clinical prognosis for the MAGL^high^ group was markedly poorer than that for the MAGL^low^ group in the 1-, 3-, and 5-year overall survival times and recurrence rates of HCC patients. MAGL expression was an independent prognostic factor for both survival and recurrence after curative resection. Furthermore, the upregulation of MAGL in HCC cells promoted cell growth and invasiveness abilities, and accompanied by EMT. In contrast, downregulation of MAGL obviously inhibited these characteristics. Moreover, further investigations verified that MAGL facilitates HCC progression via NF-κB-mediated EMT process.

**Conclusions:**

Our findings demonstrate MAGL could promote HCC progression by the induction of EMT and suggest a potential therapeutic target, as well as a biomarker for prognosis, in patients with HCC.

**Electronic supplementary material:**

The online version of this article (doi:10.1186/s13045-016-0361-3) contains supplementary material, which is available to authorized users.

## Background

Hepatocellular carcinoma (HCC) is the fifth most common malignancy and the second leading cause of cancer-related deaths [1]. Tumor metastasis and recurrence is a critical contributor to the adverse prognosis [2, 3]. Thus, a better understanding of biological characteristics in HCC becomes urgently needed. Epithelial-mesenchymal transition (EMT) is a program playing a vital role in normal biological processes including embryogenesis, tissue remodeling, and wound healing [4, 5], which also correlated with the acquisition of increased aggressive and metastatic traits of tumor cells [6, 7]. To date, increasing EMT-related transcription factors, such as Snail, Twist, and Zeb1/2, are evidenced to be required in the EMT trigger in tumor progression [8–11]. Nevertheless, the molecular mechanisms for the upstream of these factors in HCC progression are not fully elucidated. Therefore, uncovering the specific regulation mechanisms of these factors may provide a new insight in therapeutic strategies of HCC [12, 13].

Monoacylglycerol lipase (MAGL) is a lipolytic enzyme of lipid metabolism that catalyzes the conversion of monoacylglycerides to free fatty acids (FFA) and glycerol. MAGL plays a key role in several physiological processes including pain and nociperception through hydrolysis of the endocannabinoid 2-arachidonoylglycerol [14, 15]. Furthermore, MAGL also contributes to tumorigenesis and metastasis. Emerging studies have identified that expression of MAGL is elevated in many types of cancers, including melanoma, ovarian, and breast cancer [16]. MAGL could facilitate cancer cell proliferation and aggressiveness through the production of signaling lipids including monoacylglycerol (MAGs), FFA, and secondary lipid metabolites (especially LPA/PGE2). In addition, MAGL was found to be part of a gene expression signature that contains many markers of EMT [17, 18]. Indeed, EMT-related markers are predictors for increased invasion, metastasis, and poor prognosis in some human tumor types [19]. However, little is known about the physiological role of MAGL in human HCC progression.

Here, we investigated the expression of MAGL in human HCCs and its clinical significance. In particular, we explored the roles of MAGL in the growth and invasiveness of HCC cells in vivo and in vitro. We also verified the signaling pathway by which MAGL promotes HCC cell aggressiveness. Our findings provided the underlying mechanism of MAGL in progression of HCC and suggesting MAGL as a potential therapeutic target for HCC.

## Results

### Expression of MAGL is upregulated in HCC patients and associated with recurrence

**Fig. 1:**
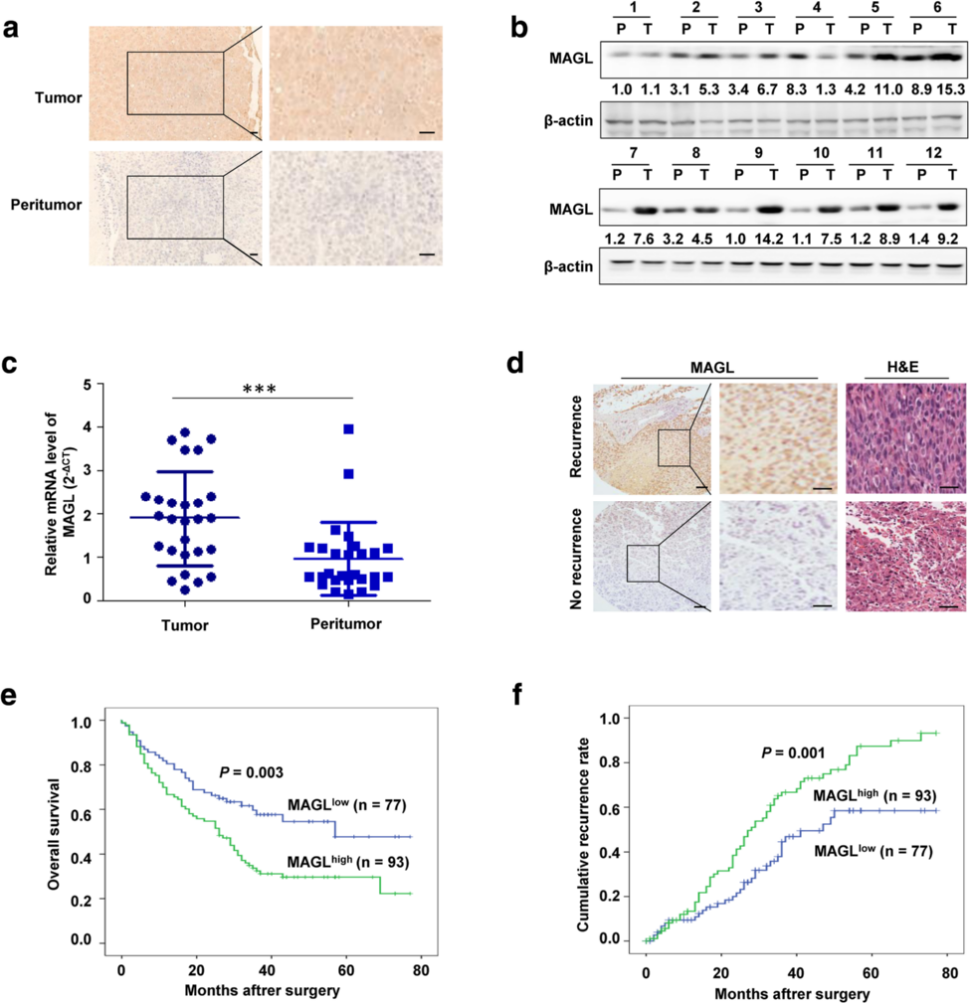
Expression of MAGL is upregulated in HCC patients and its prognostic significance. **a** Representative immunostaining images of MAGL levels in HCC tissues and corresponding peritumor tissues. *Scale bar*, 50 μm. MAGL levels were markedly higher in tumor tissues than in peritumor tissues. **b** Western blot analysis of MAGL levels in HCC tissues and corresponding peritumor tissues. **c** The expression of MAGL in 27 tumor samples were analyzed by qRT-PCR analysis. The average mRNA level of MAGL was significantly higher in tumor tissues than in peritumor tissues. **d** Immunohistochemical staining analyses of MAGL expression in 170 HCC patients and representative images are shown. *Scale bar*, 50 μm. **e**, **f** Kaplan–Meier analysis for overall survival and time to recurrence of HCC patients according to MAGL expression. MAGL overexpression in HCC predicts poorer overall survival times and recurrence rates. Data, mean + SD. ***P < 0.001

To investigate the effects of MAGL in HCC, we first detected MAGL expression in 27 tumor samples by immunohistochemical assay and found that MAGL expression was obviously elevated in HCC tissues compared with paired peritumor tissues (Fig. 1a). Consistently, western blot and qRT-PCR analysis of the indicated HCC patients demonstrated that the average expression of MAGL on both protein and mRNA levels were significantly higher in HCC tissues than peritumor tissues (Fig. 1b, c). Interestingly, immunohistochemical analysis for a large cohort of HCC samples (*n* = 170) showed that the expression of MAGL was significantly elevated in HCC patients with recurrence than those without recurrence (*P* = 0.001; Fig. 1d), which indicated a potential role of MAGL in HCC progression.

### MAGL overexpression predicts a poor clinical outcome of HCC

To explore the clinical significance of MAGL expression in HCC, we further assessed the relationship between MAGL expression and the clinicopathologic characteristics in a TMA of 170 HCC patients (Table 1). Immunohistochemical analysis showed that the average intensity of MAGL expression in tumor was significantly higher than that in corresponding peritumor tissues. Notably, elevated MAGL expression was identified to be associated with larger tumor size (*P* = 0.048), microvascular invasion (*P* = 0.026), poor tumor differentiation (*P* = 0.012), and advanced TNM stage (*P* = 0.001). Whereas the other clinicopathologic characteristics, including age, gender, HbsAg, HCV, liver cirrhosis, alpha-fetoprotein, tumor number, or tumor encapsulation, showed no correlation with the expression level of MAGL in HCC.

**Table 1:** Clinical characteristics of 170 HCC patients and correlation with MAGL staining

	MAGL staining		
Variables	Low (*n* = 77) *n*(%)	High (*n* = 93) *n*(%)	*P*
Gender			
Male	67 (87.0)	78 (83.9)	0.565
Female	10 (13.0)	15 (16.1)	
Age(years)			
≤50	28 (36.4)	37 (39.8)	0.648
>50	49 (63.6)	56 (60.2)	
HBsAg			
Negative	14 (18.2)	11 (11.8)	0.244
Positive	63 (81.8)	82 (88.2)	
HCV			
Negative	74 (96.1)	91 (97.8)	0.659
Positive	3 (3.9)	2 (2.2)	
Liver cirrhosis			
No	30 (39.0)	29 (31.2)	0.289
Yes	47 (61.0)	64 (68.8)	
AFP(ng/ml)			
≤20	28 (36.4)	23 (24.7)	0.099
>20	49 (63.6)	70 (75.3)	
Tumor number			
Single	56 (72.7)	55 (59.1)	0.064
Multiple	21 (27.3)	38 (40.9)	
Tumor size(cm)			
≤5	44 (57.1)	39 (41.9)	*0.048*
>5	33 (42.9)	54 (58.1)	
Tumor encapsulation			
Complete	37 (48.1)	49 (52.7)	0.547
Incomplete	40 (51.9)	44 (47.3)	
Microvascular invasion			
Absent	48 (62.3)	42 (45.2)	*0.026*
Present	29 (37.7)	51 (54.8)	
Edmondson grade			
I–II	16 (20.8)	36 (38.7)	*0.012*
III–IV	61 (79.2)	57 (61.3)	
TNM stage			
I	18 (23.4)	5 (5.4)	*0.001*
II–III	59 (76.6)	88 (94.6)	

*HBsAg* hepatitis B surface antigen, *HCV* hepatitis C virus, *AFP* alpha fetoprotein, *TNM* tumor node metastasis, *MAGL* monoacylglycerol lipase

Italic numbers means *P* < 0.05

In the present study, all 170 HCC patients were dichotomized as MAGL^low^ expression (*n* = 77) and MAGL^high^ expression (*n* = 93), with MAGL^high^ expression accounting for 54.7% (93 of 170). Moreover, patients in the MAGL^high^ expression group exhibited shorter OS than those in the MAGL^low^ expression group (*P* = 0.003; Fig. 1e). Consistently, the 1-, 3-, and 5-year OS rates after surgery were much worse in the MAGL^high^ expression group than those in the MAGL^low^ expression group (66.7 vs. 80.5%, 32.5 vs. 57.7%, 29.7 vs. 47.8%, respectively). In addition, the TTR in the MAGL^low^ expression group was significantly lower than that in the MAGL^high^ expression group (*P* = 0.001; Fig. 1f), and the 1-, 3-, 5-year TTR rates were significantly higher in the MAGL^high^ group than in the MAGL^low^ group (13.4 vs. 9.4%, 66.7 vs. 44.6%, 87.4 vs. 58.5%, respectively). Univariate and multivariate analysis indicated that MAGL was an independent prognostic factor for both OS (HR = 1.628, *P* < 0.05) and TTR (HR = 1.593, *P* < 0.05; Tables 2 and 3). Thus, these data clearly revealed that MAGL is a valuable predictive factor for clinical outcome of HCC.

**Table 2:** Univariate analyses of factors associated with OS and TTR

	OS		TTR	
Variables^a^	HR (95% CI)	*P*	HR (95% CI)	*P*
Gender (male vs. female)	1.375 (0.751–2.518)	0.302	1.072 (0.605–1.898)	0.811
Age (≤50 vs. >50 years)	0.863 (0.577–1.289)	0.471	1.140 (0.748–1.738)	0.542
HBsAg (negative vs. positive)	1.444 (0.788–2.646)	0.234	1.833 (0.974–3.449)	*0.061*
HCV (negative vs. positive)	1.157 (0.365–3.669)	0.805	0.612 (0.150–2.503)	0.494
Liver cirrhosis (no vs. yes)	1.337 (0.865–2.067)	*0.192*	1.476 (0.947–2.299)	*0.085*
AFP (≤20 vs. >20 ng/ml)	1.408 (0.893–2.219)	*0.140*	1.005 (0.650–1.553)	0.983
Tumor number (single vs. multiple)	1.731 (1.160–2.581)	*0.007*	1.877 (1.247–2.826)	*0.003*
Tumor size (≤5 vs. >5 cm)	2.211 (1.461–3.345)	*<0.001*	1.936 (1.278–2.934)	*0.002*
Tumor encapsulation (complete vs. incomplete)	1.566 (1.049–2.338)	*0.028*	1.116 (0.744–1.676)	0.595
Microvascular invasion (absent vs. present)	1.602 (1.074–2.389)	*0.021*	1.556 (1.033–2.345)	*0.035*
Edmondson grade (I–II vs. III–IV)	1.401 (0.898–2.184)	*0.137*	1.282 (0.826–1.990)	0.268
TNM stage (I vs. II–III)	2.117 (1.026–4.369)	*0.042*	3.260 (1.423–7.468)	*0.005*
MAGL (low vs. high)	1.856 (1.220–2.823)	*0.004*	2.040 (1.331–3.128)	*0.001*

*OS* overall survival, *TTR* time to recurrence, *HR* hazard ratio, *CI* confidence interval, *HBsAg* hepatitis B surface antigen, *HCV* hepatitis C virus, *AFP* alpha fetoprotein, *TNM* tumor node metastasis, *MAGL* monoacylglycerol lipase

^a^Variables were analyzed by Cox proportional hazards regression model

Italic numbers indicates that the variables might be associated with OS or TTR were subjected to the multivariate Cox analysis with 0.2 level for entry into the model

**Table 3:** Multivariate analyses of factors associated with OS and TTR

	OS		TTR	
Variables^a^	HR (95% CI)	*P*	HR (95% CI)	*P*
HBsAg (negative vs. positive)		NA	1.342 (0.700–2.573)	0.377
Liver cirrhosis (no vs. yes)	1.278 (0.795–2.053)	0.311	1.356 (0.855–2.149)	0.195
AFP (≤20 vs. >20 ng/ml)	1.487 (0.915–2.418)	0.110		NA
Tumor number (single vs. multiple)	1.642 (1.063–2.534)	*0.025*	1.671 (1.082–2.582)	*0.021*
Tumor size (≤5 vs. >5 cm)	2.712 (1.447–5.083)	*0.002*	1.874 (1.009–3.481)	*0.047*
Tumor encapsulation (complete vs. incomplete)	1.504 (0.988–2.289)	0.057		NA
Microvascular invasion (absent vs. present)	0.711 (0.385–1.315)	0.277	0.775 (0.419–1.436)	0.418
Edmondson grade (I–II vs. III–IV)	1.574 (0.989–2.504)	0.056		NA
TNM stage (I vs. II–III)	1.023 (0.444–2.358)	0.957	1.726 (0.687–4.340)	0.246
MAGL (low vs. high)	1.628 (1.020–2.597)	*0.041*	1.593 (1.011–2.510)	*0.045*

*OS* overall survival, *TTR* time to recurrence, *HR* hazard ratio, *CI* confidence interval, *HBsAg* hepatitis B surface antigen, *AFP* alpha fetoprotein, *TNM* tumor node metastasis, *MAGL* monoacylglycerol lipase, *NA* not adopted

^a^Variables were adopted for their prognostic significance by multivariate analysis using the Cox proportional hazards regression model

Italic numbers means *P* < 0.05

### MAGL enhances HCC cell growth and invasiveness in vivo and in vitro

**Fig. 2:**
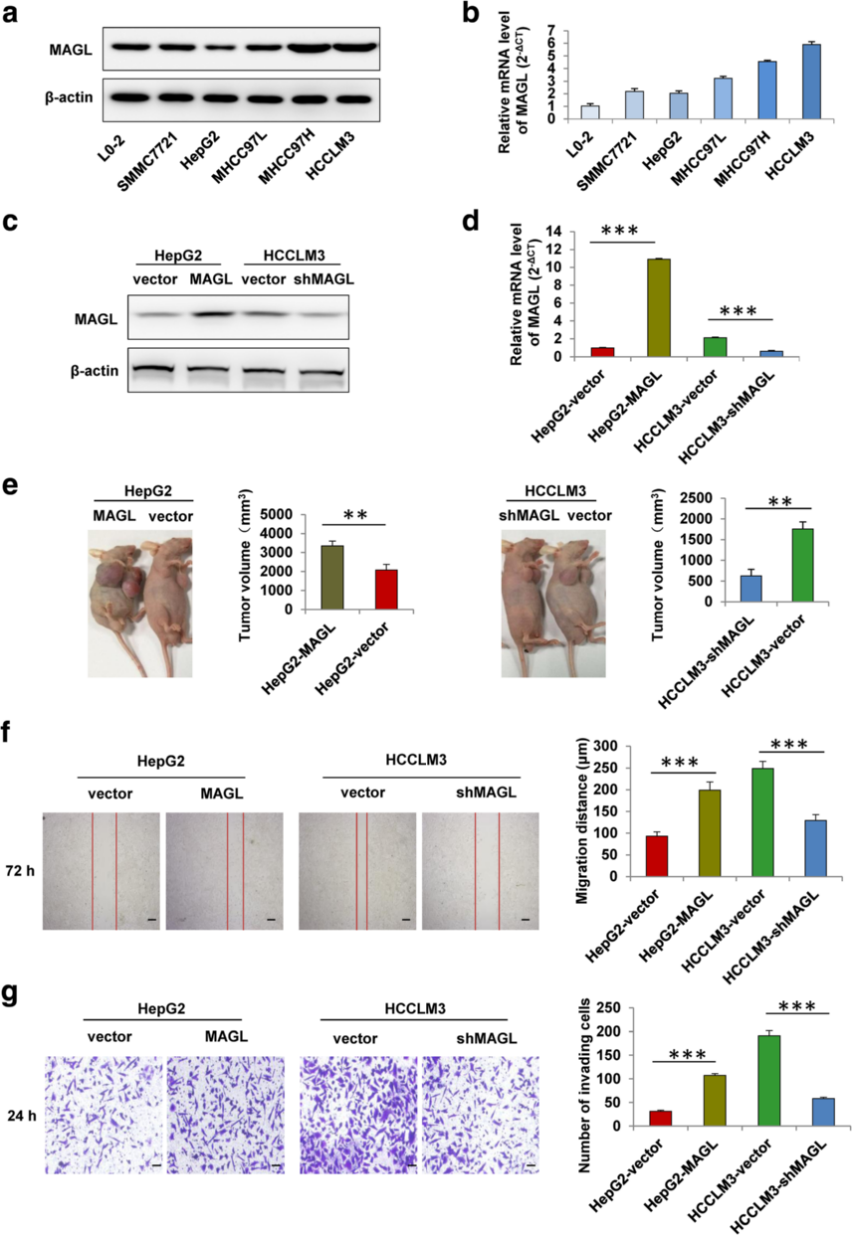
MAGL enhances HCC cell growth and invasiveness in vivo and in vitro. **a**, **b** The protein and mRNA levels of MAGL in different HCC cell lines (HepG2, SMMC7721, Huh7, MHCC97L, MHCC97H, and HCCLM3) and one normal liver cell line (L0-2). **c**, **d** Western blot and qRT-PCR analysis showed the protein and mRNA expression of MAGL in HCC cells (HepG2-vector, HepG2-MAGL, HCCLM3-vector, and HCCLM3-shMAGL). **e** MAGL overexpression promoted HCC progression in vivo. Xenograft model of HCC in nude mice were established as described in the “Methods” section. The tumor volumes of subcutaneous tumors between the experiment and control groups exhibited significant differences. **f** The motility of HCC cells was detected by scratch wound assay, and statistics are shown with a bar graph. **g** The invasion ability of HCC cells was measured by transwell Matrigel invasion assay, and statistics are shown with a bar graph. Data, mean + SD. **P < 0.01 and ***P < 0.001

To investigate the exact biological role of MAGL in HCC, we evaluated MAGL expression in various HCC cell lines (HepG2, SMMC7721, Huh7, MHCC97L, MHCC97H, and HCCLM3) and one normal liver cell line (L0-2). Data showed that the protein and mRNA expression of MAGL were significantly upregulated in HCC cell lines compared to in L0-2 cells (*P* < 0.05; Fig. 2a, b). And the expression of MAGL was enhanced in parallel with the increase of the metastatic potential of HCC cells, with the lowest level in HepG2 cells and the highest level in HCCLM3 cells. Then, MAGL expression in HepG2, a MAGL^low^ HCC cell line, was successfully upregulated (HepG2-MAGL). Meanwhile, in HCCLM3, a MAGL^high^ HCC cell line was stably downregulated (HCCLM3-shMAGL) (Fig. 2c, d). Our data showed that the growth of HepG2-MAGL cells in vivo was significantly increased (3350.0 ± 250.0 vs. 2076.7 ± 302.7 mm^3^, *P* < 0.01), whereas the growth of HCCLM3-shMAGL cells was markedly restrained (626.7 ± 157.0 vs. 1756.7 ± 172.1 mm^3^, *P* < 0.01), compared to their respective controls (HepG2-vector and HCCLM3-vector) (Fig. 2e). Moreover, the wound healing and transwell Matrigel invasion assays demonstrated that upregulation of MAGL in HepG2 cells promoted its migratory and invasive capacities, while HCCLM3-shMAGL cells with decreased MAGL levels exhibited reduced motility and invasiveness, compared with their respective controls (HepG2-vector and HCCLM3-vector) (Fig. 2f, g). Evidently, our results revealed that MAGL plays an important role in enhancing proliferative and invasive abilities of HCC cells in vivo and in vitro.

### MAGL promotes the progression of HCC by enhancing EMT

**Fig. 3:**
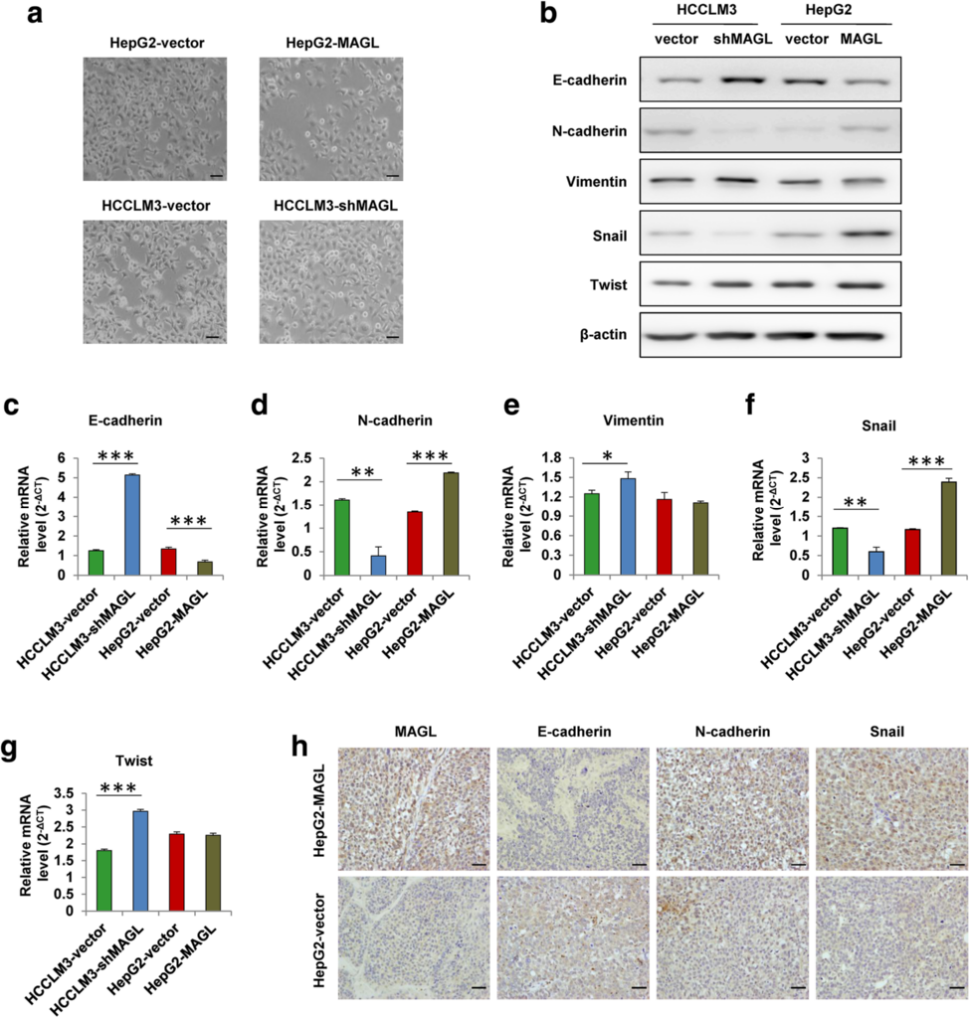
MAGL promotes the progression of HCC by enhancing EMT. **a** The cellular morphology of HCC cell lines (HepG2-vector, HepG2-MAGL, HCCLM3-vector, and HCCLM3-shMAGL). **b** Western blot assay revealed the expression of epithelial and mesenchymal markers, as well as the transcription factors, in HCC cells with different MAGL expression (HepG2-vector, HepG2-MAGL, HCCLM3-vector, and HCCLM3-shMAGL). **c**–**g** qRT-PCR analysis of E-cadherin, N-cadherin, Vimentin, Snail, Twist levels in the indicated cell lines. **h** Representative immunostaining images of EMT markers using serial sections of tumor samples. *Scale bar*, 50 μm. Data represent the mean + SD. *P < 0.05, **P < 0.01, and ***P < 0.001

To determine whether EMT contributes to the increased HCC cell growth and invasion induced by MAGL, we first identified the cellular morphology of HCC cell lines with different MAGL expression levels (HepG2-vector, HepG2-MAGL, HCCLM3-vector, and HCCLM3-shMAGL). Results showed a significant difference in morphology of the indicated cell lines (Fig. 3a). In this study, HCC cells with higher MAGL levels (HepG2-MAGL and HCCLM3-vector) exhibited a typical mesenchymal appearance which was more dispersed and presented a spindle-like morphology, while HepG2-vector and HCCLM3-shMAGL cells showed a distinct epithelial morphology which took on a clustered and cobblestone-like appearance. These results suggested that MAGL might be closely correlated with EMT process in HCC. To confirm whether EMT is essential in the enhanced invasiveness of HCC cells mediated by MAGL, we explored EMT markers by western blot and qRT-PCR analysis in the indicated HCC cells. We found that E-cadherin expression was markedly downregulated in HepG2-MAGL and HCCLM3-vector cells, while mesenchymal markers, including N-cadherin and the key EMT regulator Snail, were obviously upregulated compared with their respective controls (HepG2-vector and HCCLM3-shMAGL) (Fig. 3b–d and f). However, no significant differences were observed in other EMT markers such as vimentin and twist between groups with different MAGL levels (Fig. 3b, e, and g). Consistently, this phenomenon has also been observed in subcutaneous tumor samples by immunohistochemistry assay. Our data revealed an obvious reduction of E-cadherin expression in HepG2-MAGL group, with markedly upregulated expression of MAGL, N-cadherin, and Snail, compared to HepG2-vector group (Fig. 3h). Collectively, our data verified that MAGL overexpression might help HCC cells acquire EMT-like biochemical traits, which may contribute to MAGL-induced HCC cell growth and invasiveness.

### MAGL activated the NF-κB signal pathway in HCC cells

**Fig. 4:**
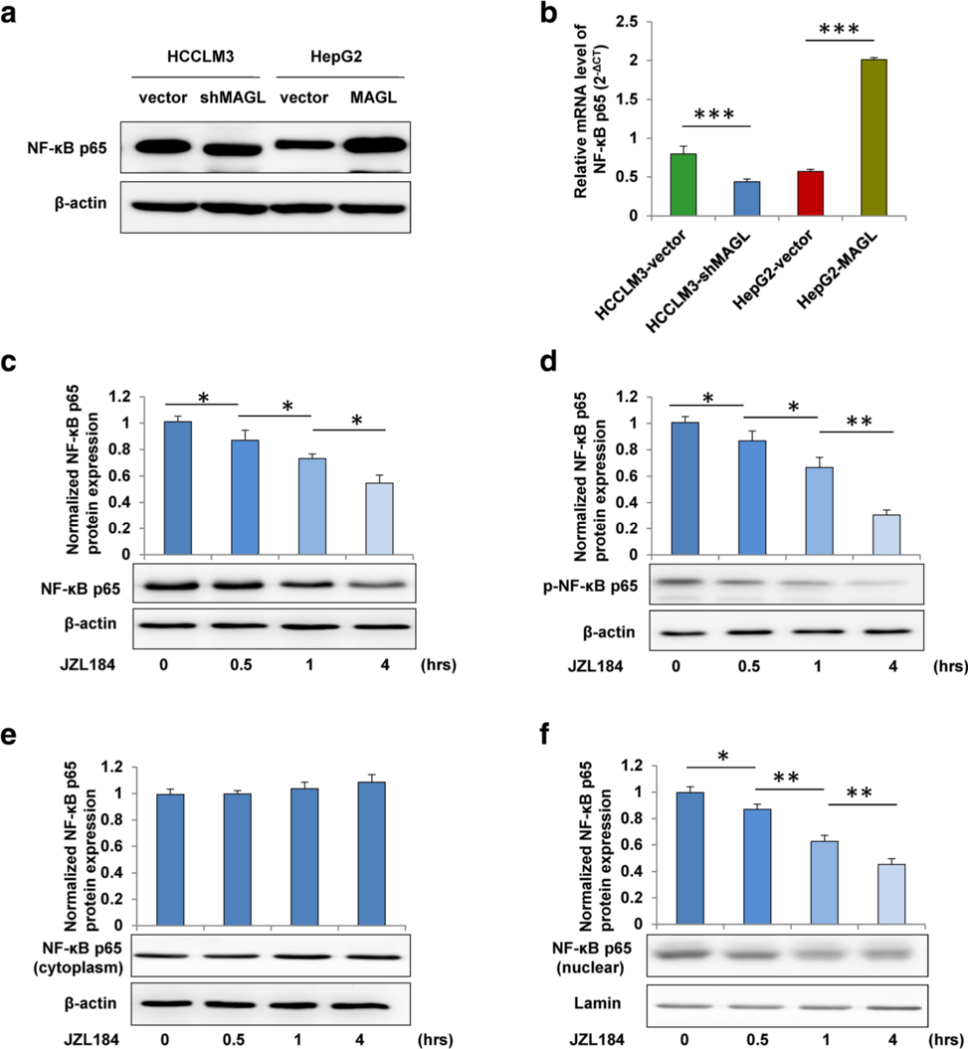
MAGL activated the NF-κB signal pathway in HCC cells. **a**, **b** The protein and mRNA expression of NF-κB p65 were evaluated in the indicated four HCC cell lines by western blot and qRT-PCR analysis. HepG2-MAGL cells were cultured with MAGL-inhibitor JZL184 (1 μM) for 0, 0.5, 1, and 4 h. **c** Western blot analysis revealed that the total protein level of NF-κB p65 was significantly downregulated in a time-dependent manner by the treatment of JZL184. **d** The phosphorylation of NF-κB p65 decreased in a time-dependent manner when treated with JZL184. **e** MAGL-inhibitor JZL184 could not inhibit the expression of NF-κB p65 in cytoplasm as culture time extended. **f** The expression of NF-kB p65 in the nucleus was significantly reduced in a time-dependent manner when treated with JZL184. Data, mean + SD. *P < 0.05, **P < 0.01, and ***P < 0.001

It is increasingly appreciated that NF-κB signaling pathway plays a vital role in the tumor EMT process [20–22]. To uncover how MAGL affects the process of EMT, we assessed the activity of EMT-related molecules by western blot analysis in HCC cells. We found that the expression of NF-κB p65 was markedly increased in HepG2-MAGL cells but significantly reduced in HCCLM3-shMAGL cells compared to their respective controls (Fig. 4a, b), suggesting that NF-κB signaling might be responsible for MAGL-regulated HCC progression. To identify this hypothesis, we treated HepG2-MAGL cells with MAGL-inhibitor JZL184 for various time periods and found the total protein expression of NF-κB p65 was downregulated in a time-dependent manner (Fig. 4c). Consistently, the expression of phosphorylated NF-κB p65 was also reduced in a time-dependent manner when treated with JZL184 (Fig. 4d), suggesting that MAGL could affect the activation of NF-κB signaling in HCC cells. Furthermore, our data showed that the level of NF-κB p65 protein in the nucleus was detected to be downregulated in a time-dependent manner, whereas this phenomenon was not observed in cytoplasm (Fig. 4e, f), suggesting that MAGL-inhibitor JZL184 could inhibit the translocation of NF-κB p65 protein into the nucleus, thus decreased the total expression level of NF-κB p65. Evidently, our results clearly displayed that MAGL-inhibitor JZL184 could inhibit upregulation, phosphorylation, and nuclear translocation of NF-kB p65 in HCC cells, which means that MAGL overexpression could promote the activity of NF-kB p65 in HCC cells.

### MAGL facilitates EMT via the NF-κB signaling pathway

**Fig. 5:**
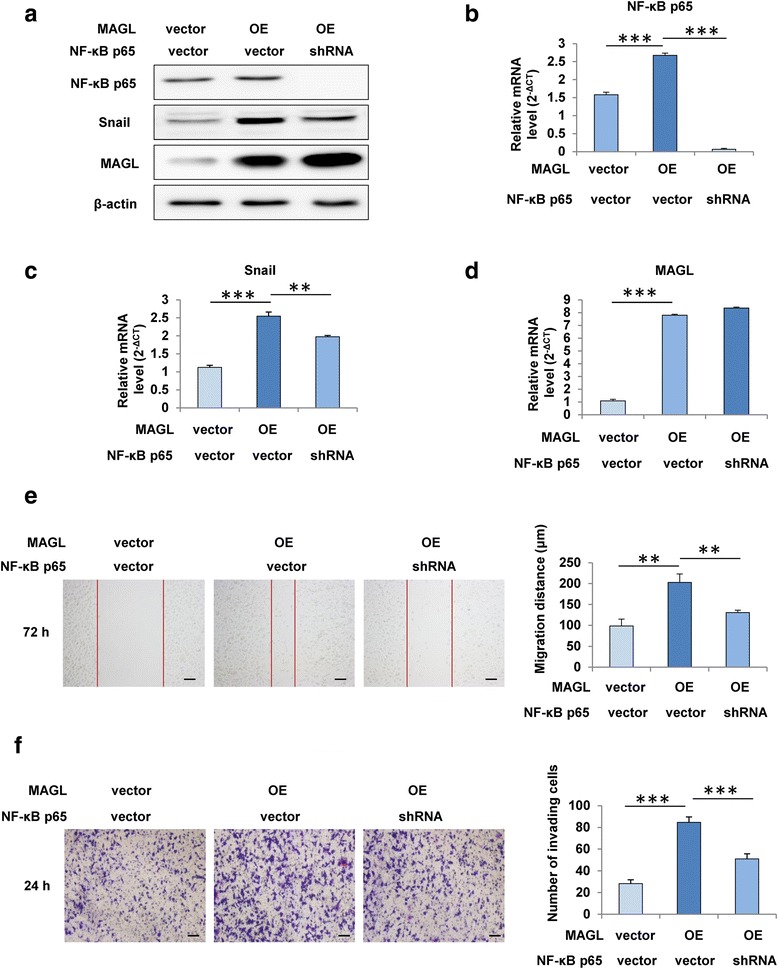
MAGL facilitates EMT via the NF-κB signaling pathway. **a** Western blot assay showed the expression of NF-κB p65, Snail, and MAGL in HCC cells with different MAGL expression (HepG2-vector, HepG2-OE). **b**–**d** qRT-PCR analysis of NF-κB p65, Snail, and MAGL expression in the indicated cell lines. **e** Scratch wound assay revealed that the blockade of NF-κB p65 in HepG2 cells by p65-shRNA significantly suppressed MAGL-induced cell migration. **f** Transwell Matrigel invasion assay indicated that NF-κB p65-shRNA treatment markedly reversed MAGL-induced invasion. Data, mean + SD. **P < 0.01 and ***P < 0.001. *OE* overexpression

To identify whether NF-κB pathway participated in the effects of MAGL on HCC cells, we blockaded the activation of NF-κB p65 using NF-κB p65-shRNA in HepG2-MAGL cells (*P* < 0.001; Fig. 5a, b). As expected, we found that the increased expression of Snail, a key EMT regulator, could markedly be reversed at both protein and mRNA levels by NF-κB p65-shRNA treatment (*P* < 0.01; Fig. 5a, c), while the enhanced expression levels of MAGL displayed no obvious changes (Fig. 5a, d). Furthermore, the wound healing and transwell Matrigel invasion assays showed that downregulation of NF-kB p65 in HepG2 cells with high MAGL expression could significantly inhibited MAGL-induced motility and invasiveness of HCC cells (*P* < 0.01; Fig. 5e, f). Collectively, these results suggested that NF-κB pathway was involved in MAGL-mediated EMT and contributed to the effects of HCC cells induced by MAGL.

## Discussion

Alterations in lipid metabolism for cancer cells are increasingly being recognized. Cancer cells require fatty acids for energy storage, membrane proliferation, and generation of signaling molecules [23]. However, less is known about the metabolic pathway that confers aggressive properties, including high migratory and invasive abilities, on malignant cancers. The present study demonstrated that MAGL is distinctly upregulated and plays a positive role in HCC progression, which might represent a new link between the metabolic signature and tumor progression.

In this study, we used a large cohort of HCC samples to show that MAGL is expressed higher in HCC tissues than in peritumor tissues, and the MAGL expression was significantly associated with larger tumor size, microvascular invasion, tumor differentiation, and TNM stage. These observations were in accordance with previous reports in other malignancies such as prostate and colorectal cancer [17, 24, 25]. Furthermore, our survival analysis indicated that HCC patients with high expression of MAGL generally had even worse prognosis than those with low expression. MAGL expression was proved to be an independent prognostic factor for both recurrence and survival after curative resection. Our findings strongly imply MAGL as a marker for HCC aggressiveness and is predictive for prognosis of HCC.

Meanwhile, we found that upregulation of MAGL could promote in vivo growth and in vitro invasiveness of HCC cells, and this process was accompanied with EMT. EMT is a program that endows tumor cells with stem-like properties, including self-renewal, enhanced survival, and anchorage-independent growth [26]. After undergoing EMT program, epithelial tumor cells could acquire enhanced invasive and metastatic traits, which was associated with high-grade malignancy. In this study, the morphological characteristics in HCC cells with MAGL overexpression transformed from epithelial to mesenchymal, and the mesenchymal markers were remarkably upregulated while the epithelial markers were significantly decreased. In contrast, HCC cells with low MAGL expression exhibited opposite features, which suggested that MAGL could enable HCC cells to acquire mesenchymal-like traits, and thus promote HCC aggressiveness. These effects have previously been reported in other tumor cell lines [27]. Our findings further confirmed the effects. Taken together, our study demonstrated that MAGL could promote HCC cell aggressiveness through, at least partially, induction of EMT.

To date, multiple signaling pathways have been evidenced to be involved in EMT process, including NF-κB, Hedgehog signaling, MAPKs, and so on. In this study, we verified that the activation of NF-κB p65 in HCC cells could be modulated by MAGL-inhibitor JZL184. Furthermore, MAGL-induced EMT in HCC cells was found to be carried out by the upregulation of Snail (Fig. 3), which depended on activation of NF-κB signaling (Fig. 5). Transcription factor Snail, which has been implicated in NF-κB- regulated EMT process [22], was evidenced to be one of the most important regulators for E-cadherin regulation [9, 10]. Thus, upregulation of MAGL in HCC cells enhanced Snail activity through the activation of NF-κB signaling, which resulted in the downregulation of E-cadherin and further triggered subsequent EMT process.

## Conclusions

In summary, we revealed a novel role for MAGL in the regulation of EMT via NF-κB pathway in HCC. Overexpression of MAGL in HCC is a potential biomarker of aggressive tumors and poor patient prognosis. Although further clinical trials are needed to assess its safety and effectiveness, our findings suggest that MAGL may be an attractive therapeutic target for HCC.

## Methods

### Patients and specimens

Tumor specimens (*n* = 27) were randomly collected from HCC patients who received curative resection at Fudan University Shanghai Cancer Center (Shanghai, People’s Republic of China). A total of 170 patients who underwent curative resection for primary HCC from 2005 to 2011 in the Liver Cancer Institute, Zhong Shan hospital, Fudan University (Shanghai, People’s Republic of China) were randomly enrolled. The detailed clinicopathological characteristics of these patients are presented in Table 1.

No patient received any preoperative anticancer treatment. Prior patient consent from each participant and ethical approval from the Research Ethics Committee of Fudan University Shanghai Cancer Center were obtained. The detailed follow-up procedures were described previously [28, 29].

### Cell lines and morphological observation

Six HCC cell lines, HepG2 (American Type Culture Collection), Huh7 (Japanese Cancer Research Bank), SMMC7721 (the Second Military Medicine College, Shanghai, China), MHCC97L, MHCC97H, and HCCLM3 (the Liver Cancer Institute of Zhongshan Hospital, Shanghai, China) were used in this study. One normal liver cell line, L0-2 (American Type Culture Collection) served as a control. All cell lines were incubated in high glucose Dulbecco’s modified Eagle medium (DMEM; Invitrogen), supplemented with 10% fetal bovine serum (FBS; Invitrogen) at 37 °C under 5% CO_2_ in humidified incubator. The morphology of HCC cells was assessed by phase microscopy (Leica).

### Quantitative real-time PCR and western blot analyses

RNA isolation, quantitative real-time PCR (qRT-PCR), and western blot analyses were performed as previously described [30–32]. The primers and antibodies used are listed in the Additional file 1: Tables S1 and S2, respectively.

### Tissue microarray and immunohistochemistry (IHC) analyses

Tissue microarrays (TMA) were constructed with the specimens collected from 170 HCC patients. Immunohistochemistry protocols were described in our previous study [33]. The immunoreactive score system (IRS) performed in this study has been described previously [34, 35]. The antibodies used in this study are listed in the Additional file 1: Table S2. The quantification of MAGL expression in TMA were evaluated by the integrated optical density (IOD) as previously described [36]. The median density of IOD was calculated and used as a cutoff value in subsequent analyses.

### Cell transfection

The short hairpin RNA (shRNA)-mediated stable silencing technique was used to assess the functional role of MAGL in HCC cells as previously described [37]. The designed target sequence for MAGL was cloned into the pLKO.1 TRC cloning vector (Additional file 1: Table S3). Lentiviral particles were constructed through cotransfection of the shRNA plasmid and the lentiviral enveloping and packaging plasmid (pMD2.G and psPAX2) into 293 T cells. The HCC cells were transfected with the viral particles, and then selected with 2 mg/ml puromycin (P8833; Sigma-Aldrich).

### Cell migration assay

Cell migration assay was performed by the scratch wound assay. HCC cells were cultured to form a tight cell monolayer, and then wounded with 200-μl plastic pipette tip. At 0 and 72 h, the migrating distance of HCC cells at the wound front were analyzed by an inverted microscope (Leica) for three randomly captured images.

### Cell matrigel invasion assay

Cell invasion assay were assessed using 24-well transwells (8 μm pore size; Corning, USA) precoated with Matrigel (Falcon354480; BD Biosciences, USA). HCC cells placed in the upper chamber were suspended in serum-free medium, and the lower chamber were added with serum-containing medium. After 24 h of incubation, the cells on the lower surface of the Matrigel membrane were fixed by paraformaldehyde, and then stained with Giemsa. The quantity of cells were counted and photographed at ×100 magnification. All experiments were carried out in triplicate.

### Animals and in vivo experiment

Male athymic BALB/c nude mice (4–6-weeks-old) were obtained from Shanghai Institute of Material Medicine, Chinese Academy of Science, and raised in specific pathogen-free conditions. Animal care and experimental protocols were performed according to the guidelines approved by the Shanghai Medical Experimental Animal Care Commission. Twenty mice were randomly divided into four groups, and 1 × 10^7^ HCC cells (HepG2-vector, HepG2-MAGL, HCCLM3-vector, and HCCLM3-shMAGL) in 0.2 ml normal saline were implanted by subcutaneous injection to construct subcutaneous tumors. Tumor dimensions were measured with vernier calipers after 4 weeks, and then calculated by the formula: tumor volume = (length × width^2^)/2.

### Statistical analysis

Data was analyzed with SPSS 19.0 for Windows (IBM) as previously described [38]. Differences among variables were compared by Pearson’s chi-square test or Fisher’s exact test. Overall survival (OS) was defined as the interval between surgery and death or between surgery and the last observation point. Time to recurrence (TTR) was defined as the interval between surgery and recurrence. The data of patients experiencing death or alive without recurrence were censored at the date of death or the last follow-up, respectively [39]. The survival and cumulative recurrence rates were performed by the Kaplan–Meier method and calculated by the log-rank test. The Cox proportional hazards regression model was used to carry out univariate and multivariate analysis. All variables might be associated with OS or TTR were subjected to the multivariate Cox analysis with 0.2 level for entry into the model. *P* < 0.05 was considered statistically significant.

## Additional file

Additional file 1: Tables S1, S2. The primers and antibodies used in the study. **Table S3.** The sequences of shRNA targeting MAGL and NF-κB p65. **Figure S1.** Representative images of MAGL immunohistochemic staining. **Figure S2.** qRT-PCR assay of MAGL mRNA expression in 27 tumor samples. **Figure S3.** The regulating effects of MAGL on NF-κB p65, N-cadherin, and Snail expression in HCCLM3 cells with different MAGL expression levels. (PDF 303 kb)

## Abbreviations

EMT: Epithelial-mesenchymal transition
HCC: Hepatocellular carcinoma
IOD: Integrated optical density
IRS: Immunoreactive score system
MAGL: Monoacylglycerol lipase
OS: Overall survival
TMA: Tissue microarray
TTR: Time to recurrence
