# Supplementary material for: Monoacylglycerol lipase promotes progression of hepatocellular carcinoma via NF-κB-mediated epithelial-mesenchymal transition
Source: J Hematol Oncol. 2016 Nov 25;9:127. doi: 10.1186/s13045-016-0361-3 (PMC5123220; doi:10.1186/s13045-016-0361-3)

Additional file 1

Supplementary Table S1. Primers used in the study

**Primers for Real-time PCR**		
**Protein**		**Sequence (5'→. 3')**
MAGL	F	ATGGTAGAGTTCGCGCCCTTGTTT
	R	TCATCATAACTCCAAGTGCTGGTCA
E-cadherin	F	TTGCTACTGGAACAGGGACAC
	R	CCCGTGTGTTAGTTCTGCTGT
N-cadherin	F	TTATCCTTGTGCTGATGTTTGTG
	R	TCTTCTTCTCCTCCACCTTCTTC
Vimentin	F	CCTTGACATTGAGATTGCCACCTA
	R	TCATCGTGATGCTGAGAAGTTTCG
Snail	F	TCCAGAGTTTACCTTCCAGCA
	R	CTTTCCCACTGTCCTACTCTG
Twist	F	GTCCGCAGTCTTACGAGGAG
	R	GTCTGAATCTTGCTCAGCTTGTC
β-actin	F	CTGAGGACAAGCCACAAGATTA
	R	ATCCACCAGAGTGAAAAGAACG

Supplementary Table S2. Antibodies used in the research

**Protein**	**Usage**	**Antibody**
MAGL	WB, IHC	Ab77398, Abcam
E-cadherin	WB, IHC	3195,CST
N-cadherin	WB, IHC	14215,CST
Vimentin	WB, IHC	5741,CST
Snail	WB, IHC	3879,CST
Twist	WB, IHC	ab50887, Abcam
NF-κB p65	WB, IHC	8242,CST
β-actin	WB, IHC	3700,CST

Abbreviations: WB, western blot; IHC, immunohistochemistry

Supplementary Table S3. Sequences of shRNA targeting MAGL and NF-κB p65

	**Sequence (5’→. 3’)**
MAGL-shRNA1	GCTCGGATGCTGATGGGGCTGGACCTGCTGGTGTTCGGGAAA<br>GCAATAGTGAAGCCACAGATGTATTGCTTTCCCCCACGACCAT<br>GTTGGCCACGGACAGAGCGAAGGGGAGAGGATGGTAGTGTCT
MAGL-shRNA2	GCTCGGATGCTGATGGGGCTGGACCTGCTGGTGTTAAATTAGT<br>GAAGCCACAGATGTAATTTATCGCCCACGACCATGTTGGCCAC<br>GGACAGAGCGAAGGGGAGAGGATGGTAGTGTCT
NF-κB p65-shRNA1	TGCTGTTGACAGTGAGCGCGGGAATCCAGTGTGTGAAGAATAG<br>TGAAGCCACAGATGTATTCTTCACACACTGGATTCCCATGCCTA<br>CTGCCTCGGA
NF-κB p65-shRNA2	TGCTGTTGACAGTGAGCGACTGAGTCAGATCAGCTCCTAATAG<br>TGAAGCCACAGATGTATTAGGAGCTGATCTGACTCAGCTGCCT<br>ACTGCCTCGGA

Supplementary Figure S1

Representative images of MAGL immunohistochemic staining. MAGL expression at low or high levels were detected in HCC samples which includes both tumor and peritumor tissues (×200). Positive cells were stained brown. Nucleus were stained blue with hematoxylin. The frozen sections of HCC samples generated from MAGL low or high expression were also stained with H&E.

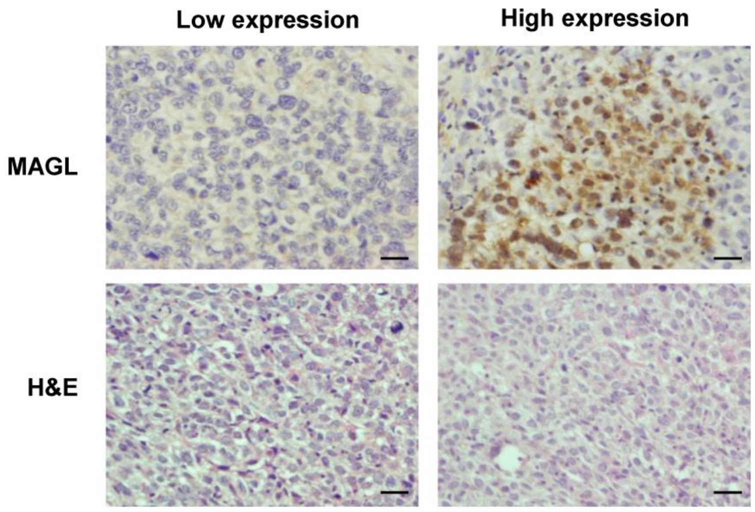

Supplementary Figure S2

qRT-PCR assay of MAGL mRNA expression in 27 tumor samples. The average mRNA level of MAGL was significantly higher in tumor tissues than in paired peritumor tissues.

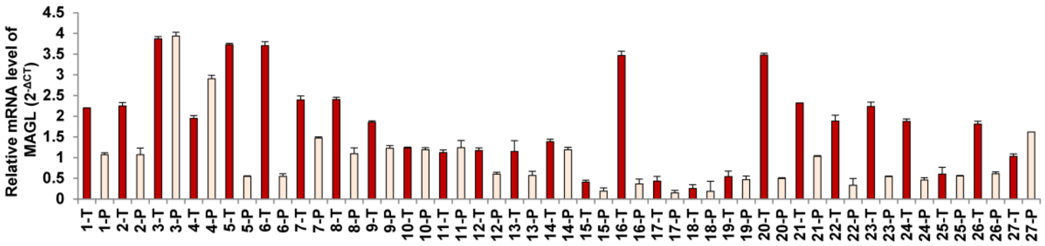

Supplementary Figure S3

The regulating effects of MAGL on NF-κB p65, N-cadherin, and Snail expression in HCCLM3 cells with different MAGL expression levels. Western blot assay showed that the reduced protein expression of NF-κB p65, N-cadherin, and Snail in HCCLM3-shMAGL cells could obviously be reversed when the MAGL expression was markedly up-regulated, which also accompanied by down-regulation of E-cadherin expression. Abbreviation: OE, overexpression.

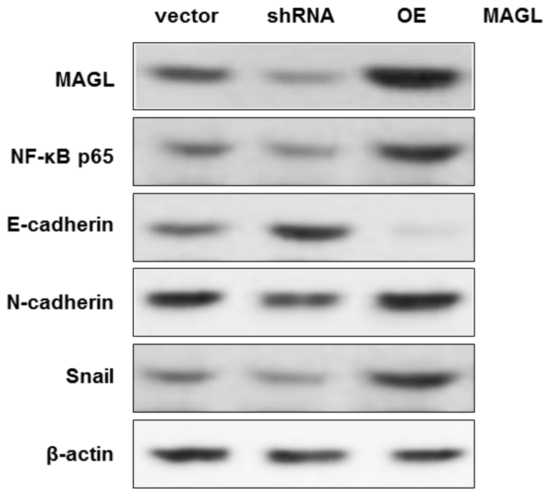

Supplement: Additional file 1: Tables S1, S2. — The primers and antibodies used in the study. Table S3. The sequences of shRNA targeting MAGL and NF-κB p65. Figure S1. Representative images of MAGL immunohistochemic staining. Figure S2. qRT-PCR assay of MAGL mRNA expression in 27 tumor samples. Figure S3. The regulating effects of MAGL on NF-κB p65, N-cadherin, and Snail expression in HCCLM3 cells with different MAGL expression levels. (PDF 303 kb) [file 13045_2016_361_MOESM1_ESM.pdf]
